# The Werner Syndrome Protein Is Distinguished from the Bloom Syndrome Protein by Its Capacity to Tightly Bind Diverse DNA Structures

**DOI:** 10.1371/journal.pone.0030189

**Published:** 2012-01-17

**Authors:** Ashwini Kamath-Loeb, Lawrence A. Loeb, Michael Fry

**Affiliations:** 1 Department of Pathology, The Gottstein Memorial Cancer Research Center, University of Washington, Seattle, Washington, United States of America; 2 Department of Biochemistry, Rappaport Faculty of Medicine, Technion - Israel Institute of Technology, Haifa, Israel; St. Georges University of London, United Kingdom

## Abstract

Loss of Werner syndrome helicase-exonuclease (WRN) or of its homolog Bloom syndrome helicase (BLM) results in different inherited disorders. Whereas Werner syndrome is characterized by premature onset of aging and age-associated diseases, Bloom syndrome involves developmental abnormalities and increased predisposition to diverse malignancies. To identify biochemical differences between WRN and BLM that might contribute to the dissimilar outcomes of their loss, we compared their abilities to unwind and bind *in vitro* diverse DNA structures. Full-length recombinant WRN and BLM proteins expressed in and purified from Sf9 insect cells unwound to comparable extents and with similar K_m_ values partial DNA duplex, splayed arm DNA and G'2 bimolecular quadruplex DNA. However, WRN resolved bubble DNA ∼25-fold more efficiently than BLM. The two enzymes were mainly distinguished by their contrasting abilities to bind DNA. WRN bound partial duplexes, bubble and splayed arm DNA and G'2 bimolecular and G4 four-molecular quadruplexes with dissociation constants of 0.25 to 25 nM. By contrast, BLM formed substantial complexes with only G4 quadruplex DNA while binding only marginally other DNA structures. We raise the possibility that in addition to its enzymatic activities WRN may act as a scaffold for the assembly on DNA of additional DNA processing proteins.

## Introduction

Evolutionarily conserved members of the RecQ subfamily of DNA helicases participate in the maintenance of genome integrity in organisms ranging from bacteria through simple eukaryotes and up to mammals. Human cells contain five RecQ proteins; RecQ1, BLM, WRN, RecQ4 and RecQ5. Mutations in three RecQ genes *BLM*, *WRN* and *RECQ4*, engender three clinically distinct respective syndromes; Bloom (BS), Werner (WS) and Rothmund-Thomson (RTS) [1], [2]. Additionally to a conserved helicase domain shared by all five human RecQ helicases, BLM and WRN proteins also include a RecQ carboxy-terminal (RQC) domain and a helicase and RNase D C-terminal (HRDC) motif, believed to be structure-specific DNA recognition motifs (reviewed in [1], [2]. Alone among all known RecQ helicases, WRN notably possesses an N-terminal exonuclease (Exo) domain that imparts exonuclease activity on the WRN protein [3], [4].

Homozygous loss of either WRN or BLM produces two markedly different disorders. WS is distinguished by premature appearance of features characteristic of aging; arteriosclerosis, type II diabetes, osteoporosis, cataracts, greying and loss of hair and skin atrophy. WS also involves increased predisposition to cancers of mesenchymal origin [5], [6]. BS, however, presents an entirely different phenotype that includes severe growth retardation, sun-induced skin lesions, immunodeficiency and infertility in males. Prominently, BS patients are predisposed to early onset of a broad range of cancers that constitute the primary cause of death in the BS subpopulation [7], [8]. The two syndromes also differ at the cellular and molecular levels. Multiple DNA transactions are defective in WS cells. Rates of replication fork propagation and DNA extension are slowed, repair of DNA damage by some agents is defective and post synaptic resolution of DNA recombination products is faulty (reviewed in [2], [9]. BS cells are characterized by a ∼10-fold increase in the frequency of sister chromatid exchange, mostly as a result of elevated frequency of homologous recombination between sister chromatids during the S or G2 phases of the cell division cycle [7], [8], [10].

Comparative studies of biochemical features of the WRN and BLM proteins have been of limited effectiveness in expounding the bases for the highly divergent clinical, cellular and molecular consequences of their loss. Commonly with all members of the RecQ subfamily, both enzymes separate the complementary strands of duplex DNA in an ATP-dependent reaction by translocating unidirectionally (3′→5′) along one strand. Both WRN and BLM are distinctive among RecQ helicases in their ability to resolve a wide variety of DNA structures. Thus, the two enzymes can unwind 3′-tailed duplexes, bubble and splayed arm DNA structures, DNA displacement loops (D-loops), four-way Holliday junctions and quadruplex formations of guanine-rich DNA [11], [12], [13], [14]. However, limited differences in the relative *in vitro* unwinding efficiencies of various DNA substrates by the two helicases [11] have thwarted attempts to pinpoint distinct cellular DNA targets of each enzyme. Both enzymes also interact physically and functionally with diverse DNA processing proteins but a considerable overlap between auxiliary protein partners of both helicases has complicated the determination of specific cellular roles of each helicase [9]. A recent advance in elucidating a potential role for BLM is notable. BLM, but not WRN, was shown to form a multiprotein complex comprised of BLM, RMI2, RMI1 and topoisomerase IIIα. This complex is believed to function in the dissolution of double Holliday junction structures and the resolution of converging replication forks employing the decatenation activity of topoisomerase IIIα. [15], [16], [17], [18], [19]. The specific biological contribution of the WRN-specific ATP stimulated 3′→5′ exonuclease activity is as yet unclear. Yet, the exclusive possession of an exonuclease activity suggests that the, as yet undefined, specific cellular roles of WRN are distinct from those of BLM and the other RecQ helicases.

To identify additional differentiating features of WRN and BLM we compared the abilities of each full-length recombinant protein to unwind and bind *in vitro* diverse DNA structures. We report that except for bubble DNA that was preferentially unwound by WRN relative to BLM, other DNA structures were resolved to similar extents by the two helicases. A major distinction between WRN and BLM was, however, their opposing capacity to associate directly with various DNA structures. Under conditions that were non-permissive for helicase activity WRN formed tight complexes with divergent duplex and quadruplex DNA structures. By contrast, BLM associated only marginally with all the examined DNA formations except for four-molecular G4 quadruplex DNA that it bound tightly. We raise the speculation that alongside its catalytic activities WRN, but not BLM, might serve as scaffold for the assembly of DNA processing multi-protein complexes on diverse structures of DNA.

## Materials and Methods

### DNA oligomers

Synthetic DNA oligomers were the products of Integrated DNA Technologies, San Diego CA. Stock solutions of 10 µg PAGE-purified DNA oligomers per µl of water were stored at −20°C until use.

### Formation of DNA structures

**Figure 1:**
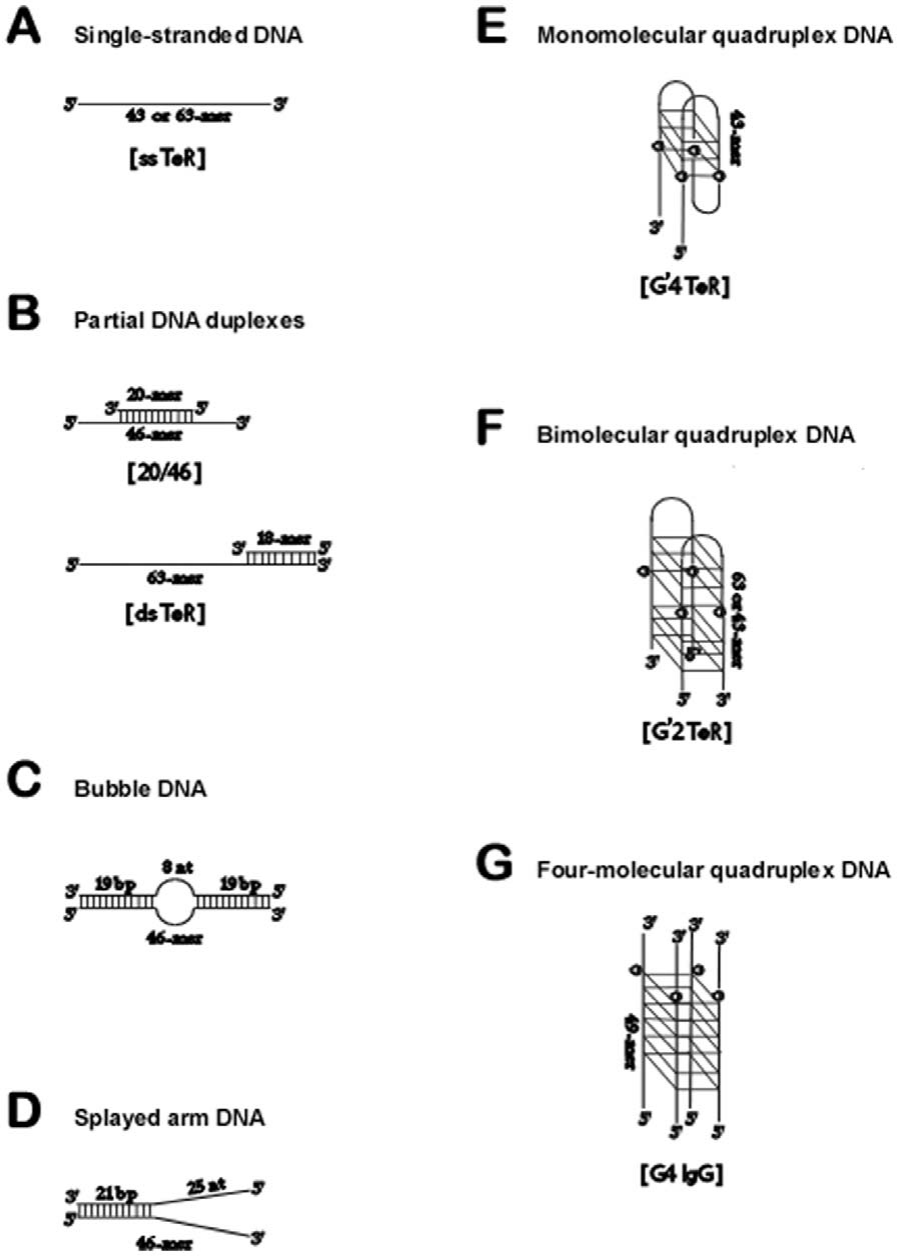
Schemes of DNA structures used in this work. Annealing of oligomers to form partial DNA duplexes, bubble and splayed arm DNA and formation of the G'4 monomolecular and G'2 bimolecular quadruplex structures of TeR_43_ and TeR_63_ oligomers and of G4 four-molecular quadruplex form of the IgG DNA switch region were performed as described under ‘Materials and Methods’.

Following dilution in water of their stock solutions, DNA oligomers were 5′-^32^P end labeled [20], ethanol precipitated and washed, dried and resuspended in indicated buffers to construct various DNA structures that are schematically illustrated in Fig. 1. Single-stranded DNA molecules (Fig. 1A) were formed by boiling for 10 min and instantly cooling to 4°C solutions in water of 1.0–2.5 µM of 43-mer or 63-mer telomeric-like DNA sequences: TeR_43_ DNA; 5′-d(GGTTAGGGTTAGGGTTAGGGTTAGGGTTAGTTAGGGTTAGGGT)-3′ or TeR_63_ DNA; 5′-d(GGTTAGGGTTAGGGTTAGGGTTAGGGTTAGTTAGGGTTAGGGTTAGGGTTAGGGGCGATTGAT)-3′. Two types of partial DNA duplexes were used (Fig. 1B): To generate a 20/46 partial duplex DNA we used 2.0 µM of an exonuclease-resistant, 3′-terminally blocked 5′-^32^P labeled 20-mer 5′-d(CGCTAGCAATATTCTGCAGC)-3′ was mixed with 4.0 µM of unlabeled 46-mer 5′-d(GCGCGGAAGCTTGGCTGCAGAATATTGCTAGCGGGAAATCGGCGCG)-3′ (complementary section underlined). Following the addition of 0.1 volume of 10 X annealing buffer, (500 mM Tris-HCl buffer, pH 8.0, 100 mM MgCl_2_) the mixture was boiled for 10 min in ∼2.0 L H_2_O and cooled slowly (4–5 h) to room temperature. The 3′-terminal inverted dC in the 5′-labeled 20-mer rendered the formed 20/46 partial DNA duplex resistant to digestion by the WRN exonuclease without affecting its helicase activity [21]. A dsTeR_63_ partial duplex was formed by mixing 75 µM unlabeled TeR_63_ DNA with 50 µM of 5′-^32^P labeled 18-mer DNA 5′-d(ATCAATCGCCCCTAACCC)-3′ that complemented the 3′-terminus of the TeR_63_ oligomer. After adding 0.1 volume of 10 X annealing buffer the mixture was boiled and cooled slowly as described above. Double-stranded DNA with an 8-nucleotide non-complementary central tract, (‘bubble DNA’; Fig. 1C) was formed by similarly annealing the 46-mers 5′-^32^P d(GCGCGGAAGCTTGGCTGCAGAATATTGCTAGCGGGAATTCGGCGCG)-3′ and unlabeled 5′-(CGCGCCGAATTCCCGCTAGTGGCGCCTTGCAGCCAAGCTTCCGCGC)-3′ (non-complementary central tract double-underlined). Splayed-arm DNA (Fig. 1D) was prepared by annealing the partially-complementary oligomers 5′-^32^P d( CGCGCCGAATTCCCGCTAGCAATATTCTGCAGCCAAGCTTCCGCGC)-3′ and 5′-d(GGCGAACTGCCATTCGCACTTACACTGCTAGCGGGAATTCGGCGCG )-3′ (complementary sections underlined). Annealing of >97% of each 5′-^32^P oligomer to its complementary unlabeled sequences was validated by non-denaturing polyacrylamide gel electrophoresis.

To generate monomolecular G'4 quadruplex structures of 5′-^32^P-labeled TeR_43_ oligomers (G'4 TeR; Fig. 1E) solutions of 1.0–2.5 µM DNA in water were boiled for 10 min, 1.0 M KCl was added to a final concentration of 100 mM and the DNA was placed on ice for 20 min. Complete conversion of the single-stranded oligomers into their respective G'4 monomolecular quadruplex structures was verified by non-denaturing electrophoresis as described [22]. Bimolecular G'2 quadruplexes of 5′-^32^P-labeled TeR_63_ or TeR_43_ oligomers (G'2 TeR; Fig. 1F) were formed by incubating at 37°C for 20–24 h solutions of 30–50 µM DNA in 100 mM KCl. Resolution by non-denaturing polyacrylamide gel electrophoresis indicated that 20–50% of the respective DNA molecules were converted into slowly migrating species whose G'2 bimolecular stoichiometry was verified as we described [23]. The remaining 50–80% of the guanine-rich DNA folded into G'4 monomolecular quadruplexes.

Formation of parallel-stranded four-molecular G4 quadruplex structure of the IgG switch region sequence 5′-d(TGGACCAGACCTAGCAGCTATGGGGGAGCTGGGGAAGGTGGGAATGTGA)-3′ (G4 IgG; Fig. 1G) and its subsequent 5′-^32^P end-labeling were conducted according to Sun and Maizels [24].

### Purification of WRN and BLM proteins

Full-length recombinant hexa-His-tagged wild-type human WRN protein, its K577M helicase-minus mutant protein and an exonuclease-minus WRN protein were purified to >90% homogeneity from Sf9 insect cells as we previously described [4]. The purified proteins were supplemented with 100 µg/ml bovine serum albumin (BSA) and stored at −80°C until used.

Full-length wild type recombinant BLM protein with a C-terminal FLAG tag was purified as follows: Sf9 insect cells (2×10^8^) that were infected with BLM-FLAG-encoding baculovirus, (a generous gift of Dr. J. D. Griffith laboratory, UNC-Chapel Hill), and harvested 48 h post-infection, lysed and the recombinant BLM protein was purified using an Anti-FLAG affinity column (Sigma-Aldrich). Briefly, insect cells were lysed in 10 column volumes of a buffer that contained 150 mM NaCl, 1 mM EDTA, 1% Triton X100, 25% glycerol, 0.2 mM PMSF and 1 µg/ml each of aprotinin, pepstatin, and leupeptin in 50 mM Tris-HCl buffer, pH 7.5. Following removal of residual particulates by centrifugation, the clarified extract was adsorbed in batch onto anti-FLAG M2 affinity gel (Sigma) at 4°C for 1 h. The resin was collected by centrifugation at 1000× g for 5 min, washed twice with 150 mM NaCl in 50 mM Tris-HCl buffer, pH 7.5 (TBS), and loaded onto a column. Resin-bound BLM protein was eluted with 5 column volumes of 100 µg/ml FLAG peptide (Sigma) in TBS and fractions were collected into tubes containing glycerol, EDTA and protease inhibitors at the above indicated final concentrations. Fractions that contained the ∼165 kDa BLM as identified by SDS-PAGE were stabilized by the addition of 100 µg/ml BSA and aliquots were stored at −80°C until use.

Approximate concentrations of the purified WRN and BLM proteins were derived from Coomassie blue-stained SDS-polyacrylamide gels with BSA as a standard.

### Assay of DNA helicases activity and determination of K_m_ values

The capacities of WRN or BLM proteins to unwind different DNA substrates were assayed and quantified by incubating stated amounts of each helicase with 5′-^32^P labeled DNA substrates in reaction mixtures that contained in a final volume of 10 µl; 4.0 mM MgCl_2_, 5.0 mM DTT, 1.0 mM ATP and 1.0 µg of bovine serum albumin (BSA) in 40 mM Tris-HCl buffer, pH 8.0. Mixtures for monitoring the unwinding of quadruplex DNA structures also contained 10 mM KCl that was necessary to prevent non-enzymatic dissociation of the tetrahelical DNA. Following incubation at 37°C for 10 min, the unwinding reaction was terminated by rapid cooling to 4°C and the addition of 2.0 µl of helicase-inactivating solution of 2.0% SDS, 50 mM EDTA, 3.0% bromphenol blue, 3.0% xylene cyanol, 40% glycerol. Unwound DNA oligomers were resolved from remaining intact DNA structures by electrophoresis at 4°C and at a constant current of up to 20–25 mA through 12% non-denaturing polyacrylamide gel (acryl/bisacrylamide, 19∶1) in 0.5 X Tris-glycine buffer, pH 8.3. Gels and running buffers that were used for the electrophoretic resolution of unwound quadruplex DNA structures were supplemented with 10 mM KCl. Electrophoresis was stopped when the bromophenol blue dye migrated 7.5–8.0 cm into the gel. The gels were dried on Whatman 3MM filter paper and exposed to Phosphor imager plates (Storm 850, Amersham Bioscience). The separated intact and unwound DNA molecules were quantified by Phosphor Imager analysis and their amounts were deduced from their measured specific radioactivity. To determine K_m_ values of the unwinding by WRN or BLM of the different DNA structures, decreasing amounts of each end-labeled DNA species were incubated at 37°C for 3 min in the described reaction mixtures. Following electrophoretic resolution of the reaction products, their amounts were quantified by Phosphor Imager analyses. K_m_ values were inferred from Lineweaver- Burk plots of results.

### Electrophoretic assay of DNA binding by WRN and BLM and determination of dissociation constants, K_d_, of the protein-DNA complexes

DNA binding by WRN or BLM proteins was conducted by incubating at 4°C for 20 min specified amounts of 5′-^32^P labeled DNA with indicated amounts of purified WRN or BLM proteins in reaction mixtures that contained in a final volume of 10 µl; 4.0 mM MgCl_2_, 5.0 mM DTT, 1.0 mM γ-S-ATP and 0.1 µg BSA in 40 mM Tris-HCl buffer, pH 8.0. Quadruplex DNA substrates were bound in mixtures that also contained 10 mM KCl to minimize non-enzymatic destabilization of the tetraplex DNA. Following incubation, protein-DNA complexes were resolved from free DNA by electrophoresis at 4°C and a constant current of up to 20–25 mA in non-denaturing 4% polyacrylamide gel (acryl/bisacrylamide, 19∶1) in 0.5 X Tris-glycine buffer, pH 8.3. Gels and running buffers that were used for the electrophoretic resolution of complexes of quadruplex DNA structures with WRN or BLM also contained 10 mM KCl. Electrophoresis was ended when a bromophenol blue tracking dye migrated 7.5–8.0 cm into the gel, the gels were dried on filter paper and exposed to Phosphor imager plates. Proportions of the free and protein-bound DNA were determined and their amounts were deduced from the known specific radioactivity of the labeled DNA probes.

To measure dissociation constants, K_d_, of DNA-protein complexes, specified constant amounts of purified WRN or BLM proteins were incubated under suitable binding conditions with decreasing amounts of 5′-^32^P labeled DNA and protein-DNA complexes were resolved from free DNA by electrophoresis in 4% non-denaturing polyacrylamide gels as described above. Following Phosphor Imager quantification of the respective bands, values of the dissociation constants, K_d_, were inferred from the negative reciprocal of slopes of Scatchard plots [25].

## Results

### Unwinding of DNA structures by WRN and BLM helicases

**Figure 2:**
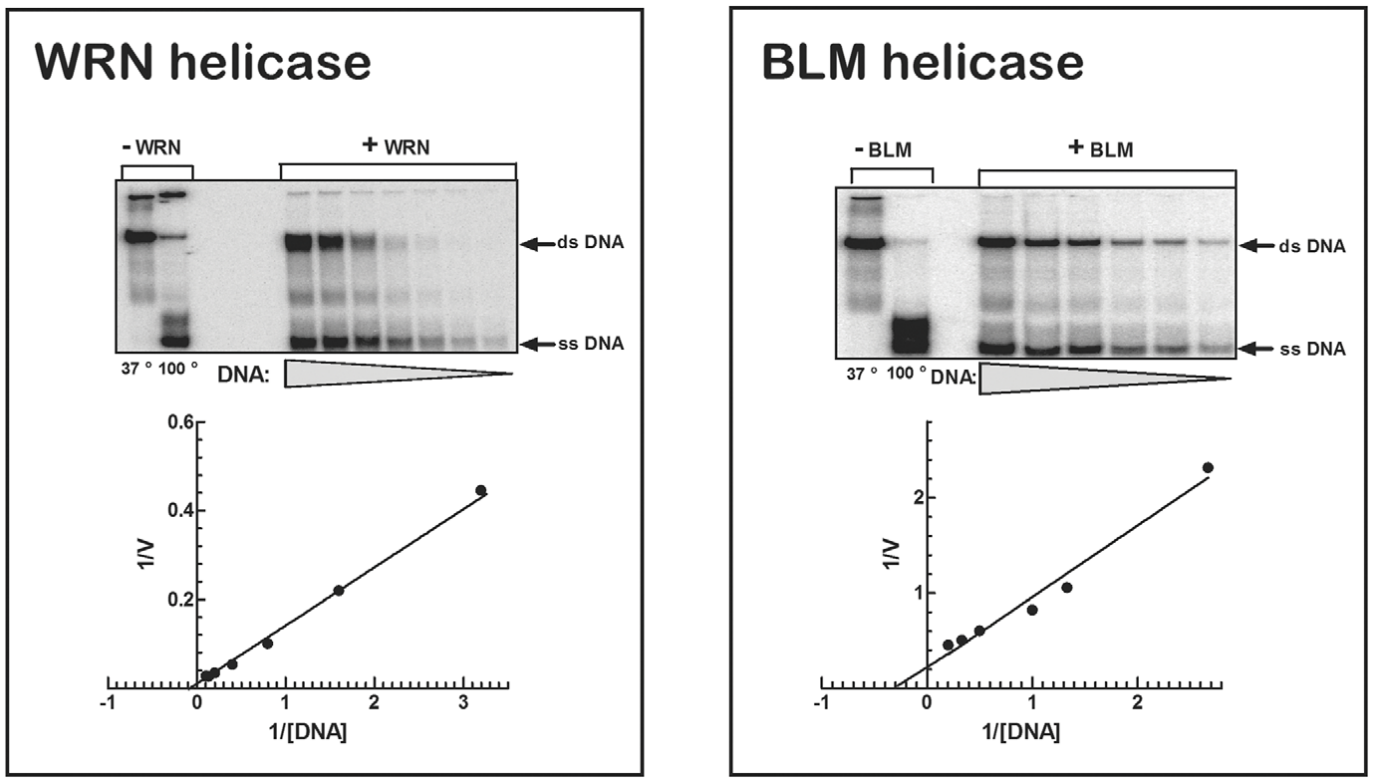
Determination of the kinetics of the unwinding of splayed arm DNA by WRN and BLM helicases. Assay conditions for helicase-catalyzed unwinding of 5′-^32^P labeled splayed arm DNA, electrophoretic resolution of unwound DNA and its quantification by Phosphor Imager analysis were carried out as detailed in the ‘Materials and Methods’ section. Upper panels; Phosphor images of splayed arm DNA resolved by electrophoresis through non-denaturing 12% polyacrylamide gels in 0.5 X Tris-glycine buffer. Controls (leftward lanes) included DNA incubated without helicase under unwinding reaction conditions and DNA boiled without helicase for 10 min to identify the position of its labeled single-strand component. Lower panels; Lineweaver-Burk plots of the quantified results.

In searching for biochemical properties that might distinguish the WRN and BLM helicases from one another, we first compared their abilities to unwind four different DNA substrates. Data presented in Table 1 indicated that WRN and BLM proteins unwound 20/46 partial duplex, splayed arm and G'2 TeR_43_ at comparable efficiencies relative to the 20/46 partial duplex (Table 1). The most prominent difference between the two enzymes was, however, their distinctly dissimilar capacities to unwind bubble DNA. Whereas WRN unwound this DNA structure at a rate that was 2.7-fold greater than that of the 20/46 partial duplex, BLM unwound bubble DNA at a rate that was only 10% that of the partial 20/46 DNA duplex (Table 1). WRN thus resolved bubble DNA at 27-fold greater relative rate than BLM. Since three out of the four examined DNA structures were unwound to similar extents by the two enzymes, we inquired whether they could be differentiated on the basis of their K_m_ values for DNA unwinding. Shown in Fig. 2 are representative electropherograms and resulting Lineweaver-Burk plots of the kinetics of resolution of splayed arm DNA by WRN and BLM. Average results of replicate similar determinations for each enzyme and DNA substrate are summarized in Table 2. The measured K_m_ values for WRN with the four DNA structures fell within a range of less than one order of magnitude, increasing in the following order; bubble DNA>20/46 partial duplex >G'2 TeR_43_ quadruplex≈splayed arm DNA. K_m_ values of BLM for the same DNA substrates except for the inefficiently unwound bubble DNA fell also within a similar narrow range as those of WRN increasing slightly in the order; splayed arm DNA>20/46 partial duplex≈G'2 TeR_43_ quadruplex. More significantly, however, K_m_ values of WRN and BLM were minimal with a maximal difference of only 3-fold for the splayed arm DNA (Table 2). Thus, the only significant difference found between the two helicases was the preferential resolution of bubble DNA by WRN.

**Table 1:** Unwinding efficacies of DNA substrates by WRN and BLM helicases.

DNA	WRN		BLM	
	Activity^a^	Relative activity^b^	Activity^a^	Relative activity^b^
20/46 partial duplex	2.2±0.1	1.0	4.8±1.3	1.0
Bubble	5.9±1.4	2.7	0.5±0.4	0.1
Splayed arm	3.5±0.7	1.6	3.5±0.9	0.7
G'2 TeR_43_ quadruplex	1.7±0.8	0.8	3.9±0.9	0.8

Increasing amounts of WRN or BLM helicases were incubated under standard DNA unwinding conditions, (‘Materials and Methods’), with 100 fmol DNA substrate per reaction mixture.

a Presented values expressed as fmols DNA unwound by 6.2 fmol helicase protein, are averages of at least three independent determinations derived from the linear sections of unwinding titration curves.

b Activity relative to the unwinding of the 20/46 partial DNA duplex.

**Table 2:** K_m_ values of the unwinding of four DNA substrates by WRN and BLM helicases.

DNA	Helicase K_m_ (N)^a^	
	WRN	BLM
20/46 partial duplex	8.9±1.9 (3)	11.3±8.0 (3)
Bubble	2.6±0.7 (3)	ND^b^
Splayed arm	17.5±5.0 (4)	5.75±2.9 (4)
G'2 TeR_43_ quadruplex	16.4±5.7 (4)	12.8±6.3 (3)

The listed K_m_ values were determined as described under ‘Materials and Methods’ and in the legend to Fig. 2.

a N – Number of independent determinations of each K_m_ value.

b ND – Not determined; the extent of unwinding of bubble DNA by BLM (Table 1) was too low to permit determination of a reliable K_m_ value.

### WRN binds a variety of DNA structures whereas BLM associates substantially with only G4 four-molecular quadruplex

**Figure 3:**
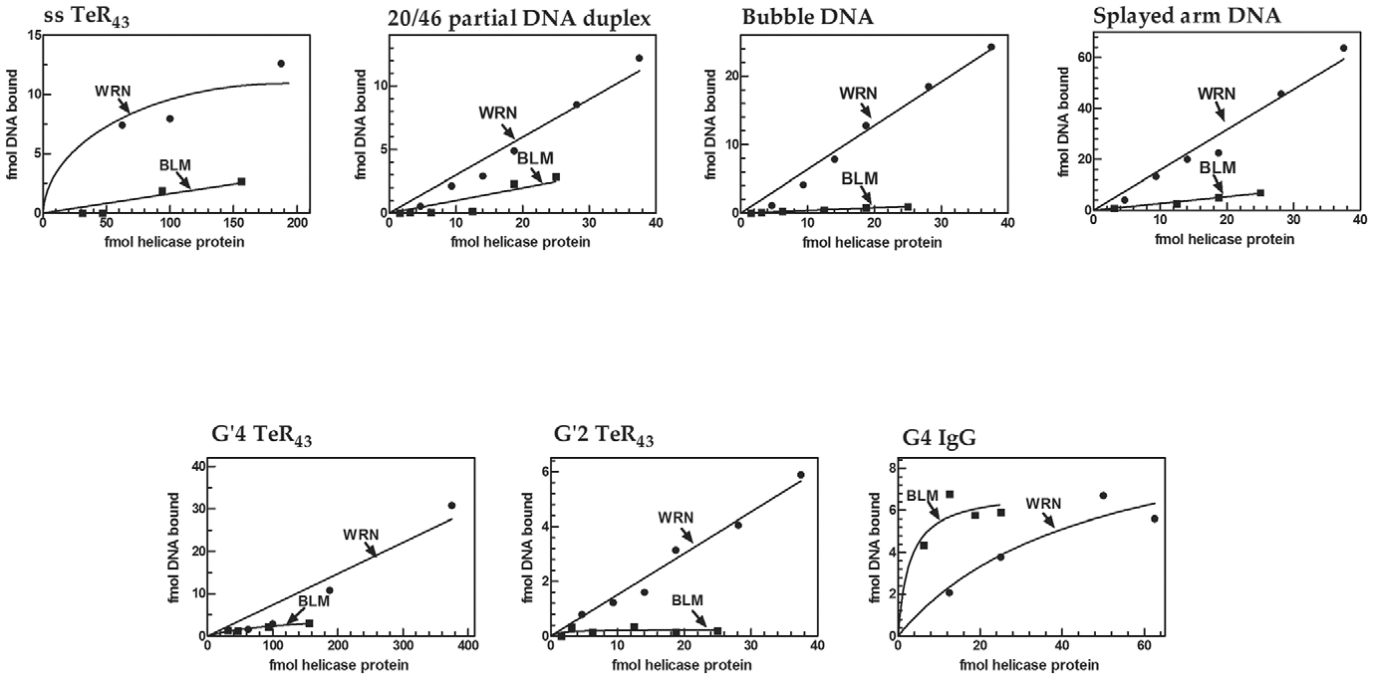
Binding of different DNA structures by increasing amounts of WRN or BLM proteins. Indicated increasing amounts of either WRN or BLM were incubated under DNA binding conditions (‘Materials and Methods’) with 120 fmol of specified 5′-^32^P labeled DNA structure per assay mixture. Protein-DNA complexes were resolved by electrophoresis through non-denaturing 4% polyacrylamide gels in 0.5 X Tris-glycine buffer with or without 10 mM KCl for quadruplex or duplex DNA, respectively. Presented are results of Phosphor Imager-quantified amounts of protein-bound DNA as a function of the amount of added helicase.

**Figure 4:**
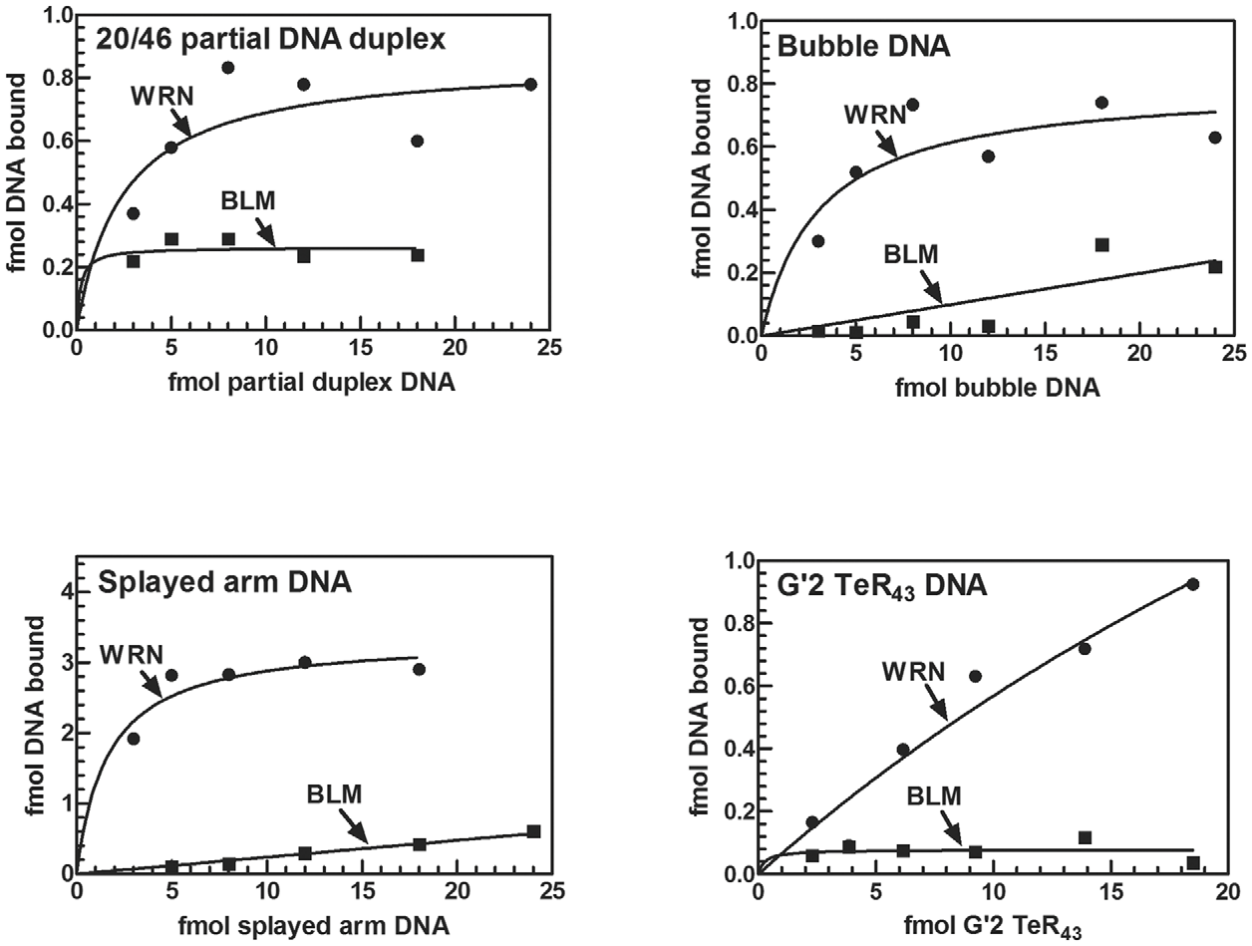
Binding of increasing amounts of different DNA structures by WRN or BLM proteins. Indicated increasing amounts of 5′-^32^P labeled specified DNA structures were incubated under DNA binding conditions (‘Materials and Methods’) with 19 or 16 fmol of WRN or BLM protein, respectively, per assay mixture. Electrophoretic resolution of the resulting DNA complexes was conducted as described in the legend to Fig. 3. Presented are results of Phosphor Imager quantified amounts of protein-bound DNA as a function of the amount of added DNA.

Since the WRN and BLM helicases did not radically differ in their abilities to unwind three of four examined DNA structures, we searched for other distinguishing properties of the two enzymes by comparing their capacity to bind various DNA conformers. End-labeled DNA structures were incubated with increasing amounts of WRN or BLM at 4°C in the presence of γ-S-ATP (“Materials and Methods”). While protein-DNA binding was enabled, both the WRN and BLM helicases were inactive under these conditions. Electrophoretic separation of protein-DNA complexes revealed that the two proteins differed greatly in their ability to associate directly with DNA. Results presented in Fig. 3 show that WRN protein substantially bound each of the examined DNA structures. By contrast, the capacity of BLM to associate with most of the examined DNA structures was marginal and it could significantly form complexes with only G4 IgG quadruplex DNA. Results presented in Fig. 3 indicated that WRN protein bound most proficiently splayed arm, bubble and 20/46 partial duplex DNA. At saturation of the DNA structures by excessive amounts of WRN, the stoichiometry of DNA to WRN protein in the complex was ∼0.5 for 20/46 partial duplex and bubble DNA and ∼1.5 for splayed arm DNA (results not shown). Formation of complexes between WRN and other DNA structures was less efficient; the stoichiometry of DNA to WRN in the complex at saturation was 0.1–0.15 for the quadruplex structures, G4 IgG and G'2 TeR_43_, and only 0.05 for single-stranded or G'4 quadruplex TeR_43_ DNA (Fig. 3 and data not shown). On the other hand, BLM protein associated proficiently only with parallel-stranded four-molecular G4 IgG quadruplex structure, reaching at saturation a stoichiometry of G4 DNA to BLM protein in the complex of ∼0.6 (Fig. 3). Little or no significant binding was observed with the remaining DNA structures such that comparable amounts of BLM associated only marginally with bubble, splayed arm or single-stranded and G'4 and G'2 quadruplex TeR_43_ DNA and were able to bind relatively low levels of the 20/46 partial DNA duplex (Fig. 3). The markedly contrasting DNA binding abilities of WRN and BLM were also observed when constant amounts of each protein were incubated with excessive amounts of 20/60 partial duplex, bubble, splayed arm or G'2 quadruplex TeR_43_ DNA. Thus, upon addition of excess DNA, WRN formed significant levels of complexes with each of the examined DNA structures whereas BLM associated only weakly with 20/46 partial duplex and bubble DNA and failed to form significant levels of complexes with splayed arm and G'2 TeR_43_ DNA (Fig. 4).

Helicase action involves alterations between ATP-bound and ATP-free forms of the enzyme. We thus inquired whether the highly dissimilar relative capacities of WRN and BLM to bind DNA structures could be due to different effects of ATP on their DNA binding conformations. Increasing amounts of either enzyme were incubated under binding conditions with 120 fmol of 5′-^32^P labeled 20/46 partial duplex with or without ϒ-S-ATP. Quantification of gel-resolved protein-DNA complexes revealed that WRN and BLM maintained their dissimilar DNA binding capacities in disregard to the presence or absence of ϒ-S-ATP. Thus, 1.0 fmol of WRN bound 0.57±0.16 or 0.45±0.15 fmol DNA with or without ϒ-S-ATP, respectively, (N = 3 for each). By contrast, 1.0 fmol of BLM bound 0.09±0.03 or 0.07±0.03 fmol DNA with or without ϒ-S-ATP, respectively, (N = 3 for each). These results indicated, therefore, that presence or removal of ATP did not alter the highly disparate DNA binding capabilities of WRN and BLM.

### Complexes of WRN with the diverse DNA structures are highly stable

**Figure 5:**
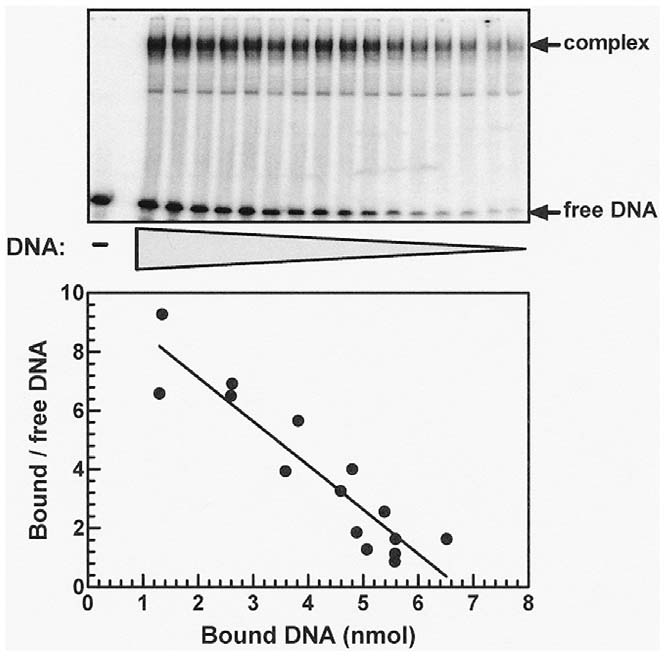
Determination of the dissociation constant, K_d_, of complexes of WRN protein with a 20/46 partial DNA duplex. Decreasing amounts of 5′-^32^P labeled 20/46 partial DNA duplex were incubated under binding conditions with 12 fmol of WRN protein per assay mixture and formed protein-DNA complexes were resolved from free DNA by non-denaturing electrophoresis through 4% polyacrylamide gels in 0.5 X Tris-glycine buffer. Upper panel; Phosphor image of gel-resolved protein-DNA complexes and free DNA. Lower panel; Scatchard plots of the quantified results. A K_d_ value was inferred from the negative reciprocal of the slope of the shown plot.

To assess the stability of complexes between WRN or BLM and different DNA structures we next determined their dissociation constants (K_d_). Levels of complexes formed by WRN with all the examined DNA species were high enough to afford reliable measurement of K_d_ values. However, the marginal binding by BLM of most of the examined DNA structures (Figs. 3 and 4) did not enable determination of K_d_ values of resulting complexes. Dissociation constants were measured, therefore, only for complexes of BLM with the substantially bound G4 IgG quadruplex. A representative gel shift resolution of complexes of WRN protein with decreasing amounts of 20/46 partial DNA duplex and Scatchard plots of the quantified results are shown in Fig. 5. Average K_d_ values of complexes of WRN or BLM with different DNA structures were acquired by similar replicate assays and analyses. Results summarized in Table 3 indicated that WRN formed stable complexes with nearly all the examined DNA structures. WRN bound most tightly bimolecular quadruplex G'2 TeR_43_ forming complexes with sub-nanomolar K_d_ values. Similarly, tight binding was measured with the longer bimolecular quadruplex G'2 TeR_63_ (results not presented). Dissociation constants of complexes of WRN with 20/46 partial duplexes, G4 IgG quadruplex and splayed arm DNA were ∼10 to 30-fold higher relative to the WRN-G'2 TeR complexes. Yet, the nanomolar range of the K_d_ values signified relative high stability of the WRN-DNA complexes. Bubble DNA that was unwound most efficiently by WRN (Tables 1 and 2) was the least tightly bound structure, forming a complex with WRN whose K_d_ was 95-fold higher than that of the WRN-G'2 TeR_43_ complex (Table 3). A notable case is that of the dsTeR_63_ partial DNA duplex that has a 5′ rather than a 3′ tail which is obligatory for WRN action. Although this structure could not serve as a substrate for the helicase (results not shown), it was readily bound by WRN - forming a complex with similar or higher stability than complexes of the helicase with its effective substrates; splayed arm or bubble DNA. This finding indicated that WRN formed complexes with DNA independent of its helicase activity. Support for this conclusion was also provided by the observation that K577M helicase-minus or exonuclease deficient mutant WRN proteins bound G'2 TeR_43_ quadruplex DNA to similar extents as the wild type protein (data not shown). This result was in line with the reported similar binding of wild type and helicase-deficient WRN to Holliday junctions that was visualized by electron microscopy [26].

**Table 3:** Dissociation constants, K_d_, of complexes of different DNA substrates with the WRN and BLM helicases.

DNA	K_d_ (nM) (N)^a^	
	WRN	BLM
20/46 partial duplex	2.3±1.3 (3)	ND^b^
dsTeR_63_ partial duplex	7.6±2.9 (4)	ND
Bubble	24.7±3.5 (3)	ND
Splayed arm	6.2±1.3 (3)	ND
G'2 TeR_43_ quadruplex	0.26±0.08 (5)	ND
G4 IgG quadruplex	3.4±0.4 (3)	7.0±2.9 (3)

The listed K_d_ values were determined as described under ‘Materials and Methods’ and in the legend to Fig. 5.

a N – Number of independent determinations of each K_d_ value.

b ND – Not determined; the extent of complex formation between BLM and these DNA structures (see Figs. 3 and 4) was too low to permit determination of reliable K_d_ values.

Of all the examined DNA substrates, BLM protein bound to a significant extent only the four-molecular, parallel-stranded G4 IgG quadruplex (Figs. 3 and 4). A measured K_d_ of 7.0±2.9 nM of the BLM-G4 DNA complex indicated that BLM protein bound this tetraplex structure as tightly as WRN (Table 3). This finding, along with the observation that BLM can unwind diverse DNA structures, suggested that the relatively weak association of BLM protein with diverse DNA structures was neither due to inactivation of its DNA binding domain nor to an inherent inability to associate with DNA. Rather, it appeared that tight binding of BLM to DNA was restricted to only the G4 four-molecular quadruplex structure (see Discussion).

## Discussion

Genetic defects in the WRN or BLM encoding genes result in distinctly different clinical, cellular, and molecular consequences. The molecular origins of these different outcomes are incompletely understood. The objective of the present work was to identify distinguishing biochemical features of WRN or BLM proteins that might be related to their specific cellular functions.

Initially, we compared the relative efficiencies of the unwinding of four DNA structures by full-length WRN and BLM helicases. Results summarized in Table 1 indicated that partial DNA duplex, splayed arm and G'2 TeR_43_ bimolecular quadruplex DNA were resolved to roughly similar extents by WRN and BLM. The comparable rates of unwinding of these substrates were also mirrored by their respectively similar K_m_ values (Table 2). The two helicases differed, however, in their capacity to resolve bubble DNA that WRN unwound at a 25-fold higher efficiency than BLM (Table 1). These results are in accord with previously reported comparable rates of unwinding by WRN and BLM of G4 quadruplex, Holliday junction and fork DNA substrates, and a ∼5-fold higher rate of resolution of bubble DNA by WRN relative to BLM [11]. The 25-fold rather than 5-fold greater efficiency of bubble DNA that we observed (Table 1) could be due to different sizes of the bubbles that were used in the two studies; 8 nucleotides (Fig. 1) versus 12 [11]. It should be noted in this context that the ability of different DNA structures to serve as substrates for the helicases is significantly affected by their length, nucleotide composition and resultant structure. Thus, whereas we reported in the past that WRN failed to unwind G'2 bimolecular quadruplex structure of a short 5-d(TAGACATGTTAGGGTTAGGGTTA)-3′ sequence [12], we showed here that WRN proficiently unwound G'2 quadruplex formations of the longer sequences 5′-d(GGTTAGGGTTAGGGTTAGGGTTAGGGTTAGTTAGGGTTAGGGT)-3′ or 5′-d(GGTTAGGGTTAGGGTTAGGGTTAGGGTTAGTTAGGGTTAGGGTTAGGGTTAGGGGCGATTGAT)-3′ (Tables 1 and 2 and accompanying text). Attempts to identify specific genomic DNA targets for WRN and BLM by measuring relative unwinding efficacies of different DNA structures *in vitro* are thwarted, therefore, by the impact of the length, sequence and structure of the DNA substrates on their accessibility to helicase action.

Our results indicated that WRN and BLM were clearly differentiated by their contrasting abilities to bind various DNA structures. Protein-DNA complexes were formed at 4°C in assay mixtures that contained non-hydrolysable γ-S-ATP in place of ATP. Neither the WRN helicase-exonuclease nor BLM helicase were active under these conditions (data not shown). DNA binding took place, therefore, while translocation of WRN or BLM along the substrate was minimized. The separation of DNA binding from the helicase and exonuclease activities of WRN, was first suggested by the equally efficient association with G'2 TeR_43_ DNA of wild type, helicase-deficient or exonuclease-minus WRN (results not presented). This disconnection was also underscored by the closely similar K_d_ values of complexes of WRN with partial duplexes that could or could not serve as substrates for helicase action. Thus, complexes of WRN with 5′-tailed dsTer partial duplex that the helicase cannot unwind had a K_d_ value of 7.6±2.9 nM, whereas its complexes with the effectively unwound 20/46 partial DNA duplex had a roughly similar K_d_ of 2.3±1.3 nM (Table 3). These independent lines of evidence support, therefore, the notion that the DNA binding function of WRN is disassociated from its enzymatic activities. Data presented in Figs. 3 and 4 demonstrated comparable efficiencies of complex formation of WRN with 20/46 partial DNA duplex, bubble and splayed arm DNA as well as G'2 bimolecular TeR_43_ and G4 four-molecular IgG DNA quadruplexes. Although WRN also bound single-stranded and G'4 monomolecular quadruplex structures of TeR_43_ DNA, the relative efficacies of complex formations with these DNA structures were ∼5–10-fold lower relative to the other DNA formations (Fig. 3). Tight binding of DNA by WRN was indicated by the nanomolar dissociation constants of complexes of WRN with the various DNA structures (Table 3). Notably, the association of WRN with G'2 TeR_43_ DNA was the tightest (K_d_∼0.25 nM) whereas complexes with bubble DNA which the helicase unwound most efficiently (Tables 1 and 2), had a 100-fold higher K_d_ of ∼25 nM (Table 3).

The efficient and tight binding of DNA by WRN contrasted the poor ability of BLM to associate with all but one examined DNA structure. Relative to WRN, BLM displayed 4 to >20-fold lower efficiencies of complex formation with 20/46 partial DNA duplex, bubble and splayed arm DNA, single-stranded and G'4 unimolecular and G'2 bimolecular quadruplex forms of TeR_43_ DNA (Figs. 3 and 4). Having been implicated in the resolution of homologous recombination intermediates, we also examined the relative capacities of WRN and BLM to bind *in vitro* Holliday junction DNA. We found that similarly to most of the examined DNA structures, Holliday junction DNA was bound by BLM at >20-fold lower efficacy than by WRN (data not shown). The only DNA structure that was effectively bound by BLM was the parallel-stranded four-molecular G4 IgG DNA quadruplex (Fig. 3, Table 3). The observed binding preference of BLM for this quadruplex structure was in line with previously published results. The Maizels laboratory reported that full-length BLM protein that was expressed and purified from yeast cells unwound and bound G4 DNA more efficiently than Holliday junction DNA [27]. A K_d_ of 4 nM for the BLM-G4 DNA complex that was measured by these authors was close to the dissociation constant of 7.0±2.9 that we determined (Table 3). A similar K_d_ value was also reported for an isolated RQC domain of BLM that was identified as the conserved G4 DNA binding site of this helicase [28]. Based on these findings and on the participation of BLM in protein complexes that appear to recognize abnormal and damaged DNA structures [15], [16], [29], [30] it was suggested that the preferential binding and unwinding of G4 DNA by BLM reflected its *in vivo* role in unwinding topological obstacles to replication [27]. Yet, side-by-side with its tight binding to G4 DNA, BLM was incapable of forming robust complexes with other DNA structures including G'2 bimolecular quadruplexes of telomeric-like sequences (Figs. 2 and 3). The highly divergent structures of the G4 and G'2 DNA quadruplexes might explain their contrasting ability to form complexes with BLM. Whereas G4 DNA is a composed of four strands arranged in parallel, the G'2 DNA structure is comprised of two antiparallel strands. In addition, variation in the number and size of hydrogen bonded guanine quartets and of non-bonded spacer sequences might also contribute to the preferential binding of G4 DNA by BLM. To accomplish catalysis, BLM must bind to its DNA substrate. The observed ability of BLM to unwind several poorly bound DNA substrates (Tables 1 and 2) suggests that its weak association with these DNA structures suffices for the execution of their resolution. In addition, it might be that auxiliary interacting proteins act *in vivo* to modulate the interaction of BLM with the DNA substrates. The tight association of BLM with G4 DNA (Fig. 3, Table 3) may indicate that BLM specializes in the selective identification and perhaps subsequent processing of parallel-stranded four- molecular G4 quadruplex structures that are thought to form in the course of recombination events such as IgG class switching.

It is tempting to speculate that the capability of WRN to efficiently form tight complexes with a variety of DNA structures independently from its helicase or exonuclease activities reflects a non-enzymatic role of this protein. The physical and functional interaction of WRN with multiple DNA processing proteins (reviewed in [9]) suggests that it acts as a component in multiprotein complexes. It might be, therefore, that in binding directly to specialized or abnormal formations in genomic DNA, WRN serves as a scaffold upon which other protein constituents of DNA processing complexes are assembled. Thus, WRN can identify and recruit other proteins to replication forks, origins of replication or replication intermediates that were modeled in this study by splayed arm, bubble and partial duplex DNA, respectively. Intriguingly, WRN protein binds most tightly to bimolecular quadruplex structures of telomeric-like sequences (Table 3). This could imply that paired telomeric ends might serve as preferential targets for WRN and subsequently for its associated proteins.
